# Mechanisms and Future of Non-Small Cell Lung Cancer Metastasis

**DOI:** 10.3389/fonc.2020.585284

**Published:** 2020-11-11

**Authors:** Tianhao Zhu, Xunxia Bao, Mingyu Chen, Rui Lin, Jianan Zhuyan, Timing Zhen, Kaichen Xing, Wei Zhou, Sibo Zhu

**Affiliations:** ^1^ School of Life Sciences, Fudan University, Shanghai, China; ^2^ Shanghai Starriver Bilingual School, Shanghai, China; ^3^ Cinoasia Institute, Shanghai, China; ^4^ Department of Neurosurgery, Huashan Hospital, Shanghai, China; ^5^ Department of General Surgery, Tongji Hospital, School of Medicine, Tongji University Medical School, Shanghai, China; ^6^ Department of Emergency, Souths Campus, Renji Hospital, School of Medicine, Shanghai Jiao Tong University, Shanghai, China

**Keywords:** non-small cell lung cancer, metastasis, treatment, mechanism, systematic literature review

## Abstract

Lung cancer, renowned for its fast progression and metastatic potency, is rising to become a leading cause of death globally. It has been long observed that lung cancer is particularly ept in spawning distant metastasis at its early stages, and it can readily colonize virtually any human organ. In recent years, cancer research has shed light on why lung cancer is endowed with its exceptional ability to metastasize. In this review, we will take a comprehensive look at the current research on lung cancer metastasis, including molecular pathways, anatomical features and genetic traits that make lung cancer intrinsically metastatic, as we go from lung cancer’s general metastatic potential to the particular metastasis mechanisms in multiple organs. We highly concerned about the advanced discovery and development of lung cancer metastasis, indicating the importance of lung cancer specific gene mutations, heterogeneity or biomarker discovery, and discussing potential opportunities and challenges. We will also introduce some current treatments that targets certain metastatic strategies of non-small cell lung cancer (NSCLC). Advances made in these regards could be critical to our current knowledge base of lung cancer metastasis.

## Introduction

Lung cancer is notoriously known for its ability to spread readily in its early stages, as well as its potency to spread to a wide range of organs of vastly different anatomy and physiology. Since lung cancer patients are often diagnosed at an advanced stage, multiple metastases would have already developed, making targeted therapy extremely difficult and systemic therapy less effective. In recent years, studies conducted to uncover mechanisms behind lung cancer are rapidly increasing in numbers.

We have learned that lung cancer cells often resist or even thrive under hypoxia, immune cells may be inactivated and manipulated by tumor cells, tumor cells can migrate through various ways, and the different mechanisms underlying lung metastasis of nearly every possible site. All the evidence piece together to reveal how complicated the concept of lung cancer metastasis is due to the numerous pathways involved in the process. In this review, we will discuss the general mechanisms of metastasis including hypoxia, immunocompromise, tumor cell migration. We shall track non-small-cell lung cancer from its primary tumor to its metastasis in the brain, the bone and the liver, which are the three most frequent sites of lung cancer metastasis. Pathology, molecular pathways and genetic characteristics of lung cancer metastasis to these organs form the bulk of our discussion. While current work of lung cancer metastasis will be reviewed with considerable depth, inferences and connections will also be frequently made to propose possible patterns of lung cancer metastasis. This review shall provide updated progress of lung cancer metastasis research, and give directions about relevant further studies.

## Results

### General Metastatic Potential

Once a cancerous cell mass has established itself, it must address a variety of environmental stress in order to support its rapid expansion as well as initiating processes that can potentially lead to metastasis. Such environmental stress includes a hypoxic stroma, a multitude of immune responses, and antagonistic local cell types that hinders its invasion. Then, tumor cells need to intravasate and survive the circulation to spawn metastasis in various organs. Through manipulation and cooperation, lung cancer cells are able to attain resistance to its hostile surroundings, even converting certain negative influences into signals beneficial to its development, enemy into allies.

#### Hypoxia Resilience

Cancer cells, being relatively metabolically active, have a certain oxygen demand. If a tumor tissue is deprived of oxygen for a prolonged period of time, it could undergo necrosis. Unfortunately, due to their low level of vascularization, tumors are generally less oxygenated compared to their surrounding tissues when they first arise, which invariably leads to intratumor hypoxia. Hypoxia is one of the defining characteristics of lung cancer and indicates poor survival (1). Although low oxygen availability in tumors reduces the effect of radiotherapy, it is also a limiting factor that severely limits tumor growth. It is estimated that tumor cells can only survive within 100 to 150 μM from a blood vessel, given that cells beyond this distance often suffer necrosis (2). To overcome this limitation, lung cancer cells use several regulation mechanisms to avoid lethal hypoxia, while reaping the benefits of low-grade hypoxia which boosts metastasis potential.

An intuitive approach for tumors to obtain adequate oxygen supply is by improving vascularization within the tumor mass through the process of angiogenesis. Several factors that facilitate this process has been identified, some of which are frequently associated with lung cancer. Vascular endothelial growth factors (VEGFs), for instance, can be secreted by tumor cells to stimulate blood vessel formation within the tumor mass. It was observed that hypoxia inducible factor (HIF) 1α expression is positively correlated with VEGF expression, and the upregulation of HIF1α and VEGF often implies poor prognosis in liver cancer (3). Discovered in 1992, HIF has been put under the spotlight as 2019 Nobel Prize in Physiology or Medicine had recently been revealed, and emerges as a promising target for cancer therapy. Among the subtypes of VEGFs, VEGF-A is currently the most extensively studied and well understood. While it can form vasculatures in tumors to compensate for the oxygen demand, newly formed blood vessels are often abnormal in shape and function. Vessels are not neatly divided into arterioles, venules, and capillaries, and are shown to be leaky, even hemorrhagic in all stages of tumor (4). Though the tumor is only getting a suboptimal supply of oxygen and nutrition, this slightly hypoxic environment does yield benefits for tumor cells. Furthermore, since newly formed vasculature is leaky, presumably because of VEGF1-A’s participation in vascular permeability regulation, there’s an increased likelihood for tumor cells to enter these abnormal vessels early on in tumor progression, especially with increased interstitial pressure accompanied with hemorrhagic leakage. This helps explain why metastasis through blood circulation often occurs at an early stage in lung cancer.

As demonstrated above, angiogenesis can only fulfill a portion of a tumor’s oxygen demand. While tumors display significant heterogeneity concerning spatial distribution of hypoxic regions, the vast majority of tumor cells are subsisting under non-lethal hypoxia (5). This characteristic of the tumor microenvironment is shown to facilitate an array of processes contributive to enhanced metastatic potential. It is observed that Epithelial-Mesenchymal Transition (EMT) is profoundly increased in hypoxic tumor tissues, and various mechanisms had been proposed since to explain this phenomenon. Beta-catenin is found to be accumulating in hypoxic tumor cell nucleus and is pinpointed as an agent that increases the expression of EMT-related genes (6). One previous study reveals that beta-catenin enhances HIF-1 mediated transcriptions, promoting tumor survival. This study also confirms that beta-catenin signaling is aberrant under hypoxia (7). Recent studies have attempted to explain beta-catenin’s EMT inducing potential, and a link has been drawn between beta-catenin and Wnt pathway, known as Wnt/beta-catenin signaling. Through stabilizing beta-catenin and translocating it into the nucleus, hypoxia in adenocarcinoma cells in lungs eventually results in enhanced Wnt signaling activity. Research had shown that a disruption in regulated Wnt/beta-catenin (8) signaling could induce EMT in tumor cells, and it is proposed that it is caused by the loss of E-cadherin, which is detrimental to the maintenance of epithelial integrity (9). Alternative mechanisms have also been proposed. A study in 2016 concludes that PTEN phosphatase activity can be altered under persistent hypoxia. PTEN phosphatase appears to inhibit EMT in tumor progression, while phosphorylation of the PTEN C-terminus (p-PTEN) leads to diminishing activity of PTEN phosphatase. Lung cancer cells exposed to hypoxia demonstrated sharp decrease in PTEN phosphatase and increase in p-PTEN, which accompanies a high rate of EMT (10). TWIST, recently recognized as another crucial mediator of cancer metastasis, is shown to be influenced by a hypoxic microenvironment. TWIST negatively correlates with E-cadherin expression, while positively correlates with HIF1-α expression. It is considered that HIF-1α derived from hypoxic environment enhances TWIST expression (11). High-level expression of HIF-1α and TWIST, therefore, indicate increased EMT occurrence, metastasis likelihood and a poor prognosis (12). EMT grants cancer cells higher mobility and stem-cell properties which both facilitate distant metastasis. Given how fundamental EMT is to most metastasis, hypoxia evidently contributes to the metastasis potential of lung cancer, possibly through multiple signaling pathways.

### Immunocompromise

Aside from having to adapt to unfavorable oxygen status, tumor cells also need to fend off another major threat. Recognizing tumor cells as foreign, a myriad of immune cells will actively attack them upon contact, reducing the tumor’s capabilities to expand. In fact, the immune system is so efficient in performing its antitumoral functions that, if a detectable tumor does arise, its cells must have evaded local immunity (13). Tumor cells develop specialized tactics against each component of the local immune system. The following paragraphs will explore the mechanisms tumor cells use to either evade or cooperate with various immune cells.

Given the fact that cancer cells are still bodily cells in nature, they are most prone to attacks launched by members of cell-mediated immunity. One such attack is initiated by cytotoxic T cells (CD8+). CD8+ tumor-infiltrating lymphocytes (CD8+ TILs) can infiltrate into tumors and induce cytotoxic effects through secretion of cytokines, which triggers inflammation and immune responses (14). They can also secrete substances such as granzymes and perforins for immediate cytotoxic effect. Its presence is correlated with a better prognosis, but its function can be suppressed by several factors (15). Programmed death-ligand 1 (PDL1)-Programmed death receptor 1 (PD1) pathway is shown to promote dysfunction in tumor-responding T cells in lung cancer patients (16). Tumors cells expressed PDL1 can bind to PD1 on the surface of CD8 T cells, reducing its antitumoral effectiveness (17). Immunomodulation therapies targeting this interaction in non-small cell lung cancer (NSCLC) shows promise to enhance therapeutic effects (18).

Apart from directly manipulating CD8 T cell functionality, tumor cells also utilize many indirect mechanisms with the end goal of suppressing CD8 T cell activity. Dendritic cells (DC), a potent antigen-presenting cell (APC) is shown to play a role in immune suppression in lung cancer. DCs isolated from NSCLC display increased secretion of immunosuppressing molecules such as the aforementioned PDL1 (19). DCs are heterogeneous in nature, which can be subdivided into tumor-associated DCs (TADC), which orchestrates T cell antitumoral processes, and regulatory DCs, which accelerates tumor growth by immunosuppression (20). Tumor cells take advantage of DCs’ heterogeneity as well as its plasticity to alter the composition of DC pool, impairing the overall antigen-presenting ability of DCs while magnifying its protumorigenic effects (21). Research also suggests that certain regulatory DCs also inhibit helper T cells (CD4+) activity, which disable another antigen-presenting pathway, reducing CD8+ T cell activity further (22). Regulatory T cells (Tregs), which normally function as an immunosuppressing agent through expression of FOXP3, acquire enhanced effectiveness in the tumor microenvironment (TME). Similar to DCs, Tregs exhibit high heterogeneity and plasticity, constituting another pool of immune cells tumor can turn to its favor (23). Tumors seem to possess the ability to chemically attract a subtype of Tregs known as FOXP3(+)CD25(+)CD4(+) Treg, which differentiates into a subpopulation that suppresses effector T cells functions (24). This is exemplified by a study involving 92 patients with NSCLC, which discovered increased Treg activity in intratumoral regions, including the overexpression of FOXP3 mRNA (25). Increased level of FOXP3 is shown to enhance the viability and invasiveness of tumors in NSCLCs, thus Treg can be yet another link in immunity which lung cancer might exploit to promote its growth (26). In conclusion, diminishing effect of CD8+ T cells caused by various mechanisms corresponds to greater invasiveness of lung cancer, giving it access to more capillary beds in the lungs, which increases likelihood of blood mediated metastasis.

CD8+ T cells are not alone in the frontline battle against cancer. Natural Killer (NK) cells, which constitute a major part of the innate immune system, also have antitumorigenic capabilities. Unlike CD8+ T cells, which requires a round of clonal expansion before unleashing its cytotoxic effects, NK cells can directly kill tumor cells, such as through the secretion of TNFα. A study conducted on mice subjects concluded that lung resident NK cells have a dominant role in suppressing metastatic tumor growth in the lungs (27). Numerous factors had been studied to establish pathways leading to NK cell exhaustion. Prostaglandin E2 (PGE2) secreted by lung cancer cells is attributed to diminishing effect of NK cells in one study, which shows that a range of soluble factors including PGE2 can inhibit the release of perforins and granzymes produced by NK cells (28). The loss of interleukin 2 (IL2) is also considered as a suppressive factor since IL2 is critical to triggering cytolytic activities in NK cells (29). Human leukocyte antigen E (HLA-E), a ligand overexpressed in tumor cells, can bind to inhibitory receptor NKG2A which dampens NK cell response to malignant growth (30). In cisplatin-resistant lung cancer cases, a specific pathway is unmasked. Higher expression of fatty acid synthase (FASN) in cisplatin-resistant tumor cells signals the downstream molecule TGFβ1, resulting in elevated PD-L1 expression that suppresses NK cell functions (31). In short, through multiple signaling pathways, lung cancer cells evade the cytolytic effects of NK cells, potentially giving it a greater chance to survive at distant sites surveilled by NK cells.

The last type of immune cells that will be discussed in this section are macrophages. Macrophages make up the majority of tumor-infiltrating immune cells, often triggering local inflammation (32). The dual role of macrophage has been observed in tumor masses. Early on in tumor emergence, macrophages either engulf individual tumor cells or act as APCs to provoke immune response from CD8+ T cells. When CD8+ T cells eventually fail to mount an immune effect on the tumor, tumor-associated macrophages (TAMs) promote tumor growth by secreting growth factors or facilitating angiogenesis (33).

Although alternative classifications have been proposed, a binary system is still widely used, which subdivides macrophages into M1 and M2 types (34). Inhibition of NOTCH-1 is proposed as a possible pathway that enhances M2 differentiation in tumors. When notch-1 is inhibited, enhanced M2 differentiation is observed (35). One study clarifies individual effects M1 and M2 has on lung cancer development. Using lung cancer cell A549, their experiments showed that M2 subtype promotes A549 invasion, while the M1 subtype inhibits angiogenesis in tumors (36). The M2 subtype’s protumorigenic function is further proven in an experiment that uses β-elemene to inhibit M2 activity, resulting in suppressed tumor growth (37). M2 macrophages are shown to have angiogenesis effects that M1 macrophages do not possess. FGF signaling for M2a and PlGF signaling for M2c are proposed as possible mechanisms behind the angiogenic effect of M2 macrophages (38). Not only do M2 macrophages stimulate angiogenesis and suppress immune responses, they also prepare target organs for metastasis, as well as promoting intravasation of tumor cells (39). Macrophages are also associated with EMT in tumor cells, since inflammation is shown to be a key factor in inducing EMT. Given that certain macrophages, like M1 macrophages, are proinflammatory, they might increase EMT occurrence in cancerous cells (40). Another 2017 research concludes that M2 macrophages can induce EMT through the TGF-β/Smad2 signaling pathway. M2 macrophages secrete large amounts of TGF-β, which can induce EMT in lung alveolar epithelial cells. Specific changes in genetic expression include heightened α-SMA and lowered E-cadherin and CK18, which contributes to cell mobility and the loss of epithelial adhesion (41). Another Chinese team confirms that E-cadherin level does correlate with TAMs density, and reveals that TAMs promote a certain type of EMT in lung adenocarcinoma through FUT4/LeY-mediated fucosylation (42). With angiogenetic and EMT capabilities combined, macrophages are a major candidate for facilitating lung cancer growth and metastasis.

### Cell Motility and Migration

Rather than remaining stationary, many tumor cells turned to a nomadic lifestyle. Tumor cell migration often depends on the crosstalk of different mechanisms. In order to move around in its extracellular matrix (ECM), tumor cells must make changes to themselves and cooperate with other cell types present in the local microenvironment. Better cell motility grants malignant cells enhanced potency to invade normal tissues, as well as a heightened chance of entering vasculature (43).

Tumor cells are not endowed with the ability to move on themselves at an early stage. Therefore, tumor cells must undergo significant changes to enable movement. Cell membrane protrusion is one of the first characteristics of migratory tumor cells acquire. The formation of these protrusions, such as lamellipodia and invadopodia, is driven by the interaction between several proteins and cytoplasmic structures, one of them being the actin cytoskeleton (44). The actin cytoskeleton requires actin-binding proteins to function, and some of these actin-binding proteins are shown to be correlated with cancer progression. A study in 2017 discovered that cortactin, an action-binding protein, is often overexpressed in tumors. Cortactin promotes tumor cell invasiveness by forming invadopodia and degrading surrounding ECM. It is believed that the overexpression of cortactin is caused by the amplification of a chromosomal band (45). Another study postulated that since actin and actin-binding protein are observed to be accumulating in tumor cell nucleus, they may alter gene expression, which could potentially explain the genetic amplification observed in the previous study (46). Actin has been linked to the formation of multiple membrane protrusions. One research suggests that cortactin and dynamin-2 stabilize F-actin bundles in filopodia in lung cancer cell line H1299 (47). Furthermore, cortactin is also shown to participate in invadopodia formation. Inhibiting cortactin by miR-182 negatively impacted invadopodia formation (48). Cortactin is not the only protein that facilitates the acquisition of cell motility through membrane protrusions. The range of proteins participating in forming membrane protrusions is inconceivably large. Tks5, an adapter protein that contributes to invadopodia formation, is shown to have increased expression in lung carcinoma. The overexpression of Tks5 is correlated with increased metastatic potential and worsened prognosis (49). Another study in 2014 also identifies Tks5 as a critical factor for invadopodia maturation, while pointing to cortactin and Tks4 for invadopodia initiation and function, respectively (50). A study concerning invadopodia pinpointed β3 integrin as indispensable to invadopodia’s formation and its ability to degrade the surrounding ECM. Increased β3 integrin expression often indicate a greater probability of metastasis in lung cancer (51). A decrease in nexin 9 (SNX9) in invadopodia in NSCLC is correlated with tumor invasiveness. SNX9 normally inhibits invadopodia function by binding to TKS5, therefore the depletion of SNX9 result in increased degradation of ECM (52). As demonstrated above, lung cancer cells may utilize a range of protein regulation to enhance its motility through the formation of membrane protrusions.

Tumor cells can also steer cells in the ECM into their favor. Myofibroblasts (MF), which normally participate in tissue repair, may cooperate with cancer and promote tumor cell migration (53). TGF-β is shown to be involved in the creation of MF. One study established a positive feedback signaling loop formed by TGF-β and IL-6/JAK2/STAT3 signaling pathways, which mediates interactions between MF and tumor cells (54). Although TGF- β can exist in soluble form, it is also found on surfaces of exosomes produced by tumor cells, which might explain why MF presence is heightened in tumor ECM (55). Exosomal TGF- β also has greater potency in signaling myofibroblastic differentiation. When exosomes are eliminated from cancer cell secretome, myofibroblastic differentiation ceases, leading to failure of stroma-assisted tumor growth (56). MF express metalloproteinases (MMPs), which can alter matrix proteins. Studies show that MMPs are highly expressed in lung tumors and are actively involved in ECM degradation (57). A study performed on A549 NSCLC cells demonstrates that upregulation of MMP-9 promotes tumor cell migration and increases invasiveness (58). Moreover, using ECM degradation techniques such as MMPs, lung cancer cells can also degrade basement membrane of capillaries to intravasate. In conclusion, through cooperating with cells like MF, lung cancer cells can attain greater efficiency in degrading its surrounding ECM, allowing it to migrate with increased pace.

### Going Organ-Specific

**Figure 1:**
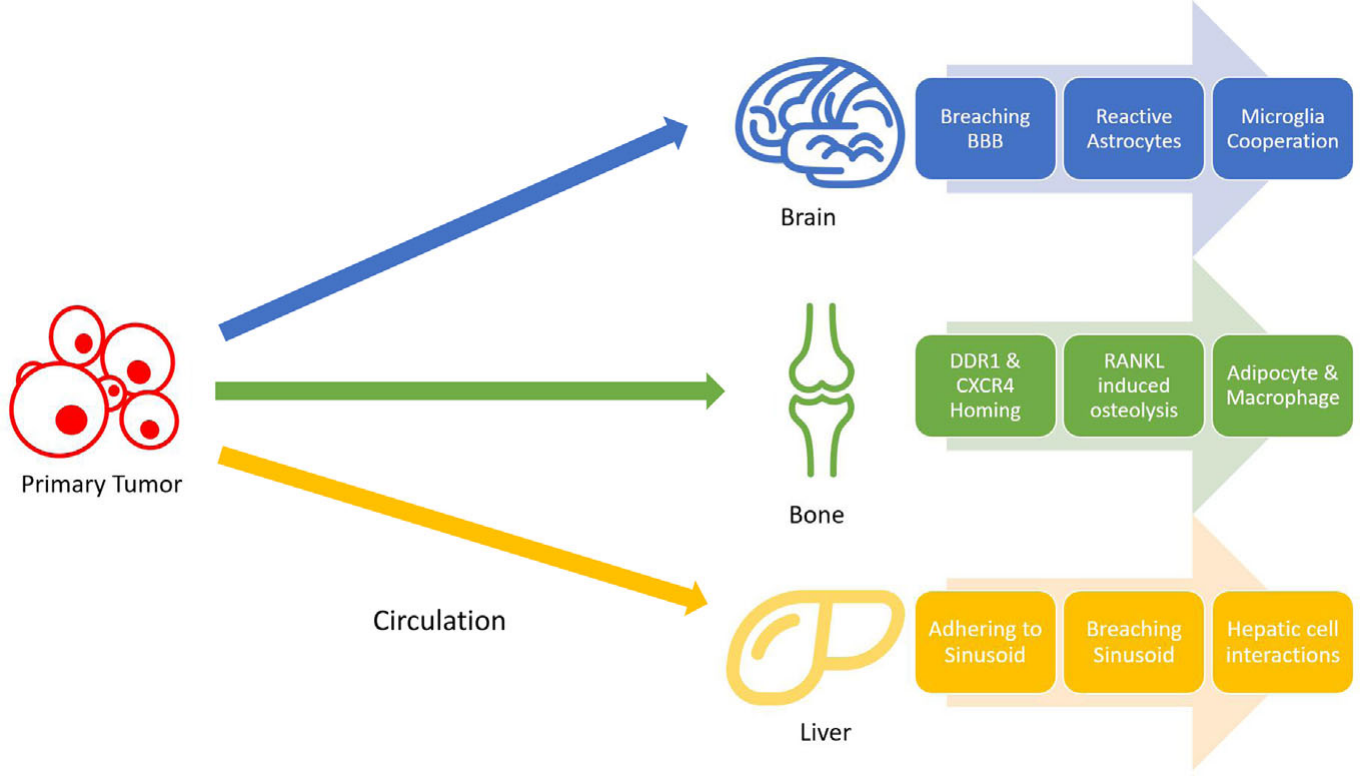
Non-small cell lung cancer (NSCLC) metastasis stages. Tumors cells gain mobility and intravasate into bloodstream, where they will travel to distant organs and extravasate. Once in the target organ tissues, tumor cells adapt to their surroundings by resisting local immune attacks and cooperating with different cells.

Lung cancer is distinctive from many other types of cancer regarding the wide range of organs it could metastasize to. The nervous system, bone, liver, respiratory system, and adrenal gland, ranked by overall incidence rate, are all colonizable by lung cancer metastasis, and such metastasis all foretell a worsened prognosis (59). Understanding the mechanisms behind such a width of potential metastasis of lung cancer can unlock new therapeutic strategies to prevent the formation of metastasis. The following sections will concern less about why lung cancer metastasize frequently in general, but rather focus more on mechanisms that facilitate metastasis formation in individual organs favored by lung metastasis. Although the colonization of tumor cells in these organs follow a similar pattern, they involve vastly different molecular pathways based on their immediate surroundings (**Figure 1**).

#### Brain

Being one of the most protected organs in the body, the brain is surprisingly prone to lung cancer metastasis. There has been some advancement on decrypting the homing mechanisms used by tumor cells. CD15 is a cell adhesion molecule found overexpressed in metastatic NSCLC cells. Its binding counterpart, E-selectin (CD62E), is also found overexpressed in hCMEC/D3 human brain endothelial cells through activation by TGF-α. This increases the chance for metastatic NSCLC cells to bind to the brain endothelium, which opens the gateway for tumor cell intrusion (60). The effect of TGF-α can be dampened by EGFR specific tyrosine kinase inhibitor (61), and is shown to be downregulated by Yin Yang 1 (YY1), a potential therapeutic target (62). CD15-CD62E interaction is exclusively found at adhesion sites of tumor cells and brain endothelium while blocking this interaction significantly reduces cancer cell’s ability to attach to the brain endothelium (63). Another study suggests that metastasizing lung cancer cells might secrete substances that damage endothelial glycocalyx. The glycocalyx serves as a protective covering of brain endothelium, which can have various adhesive interactions with tumor cells (64). its degradation is correlated with enhanced E-selectin-mediated adhesion (65). Many other cellular adhesion molecules (CAMs) have also been identified as relevant to brain metastasis, such as ALCAM/ALCAM and VLA-4/VCAM-1 interactions identified in a 2014 study (66).

**Figure 2:**
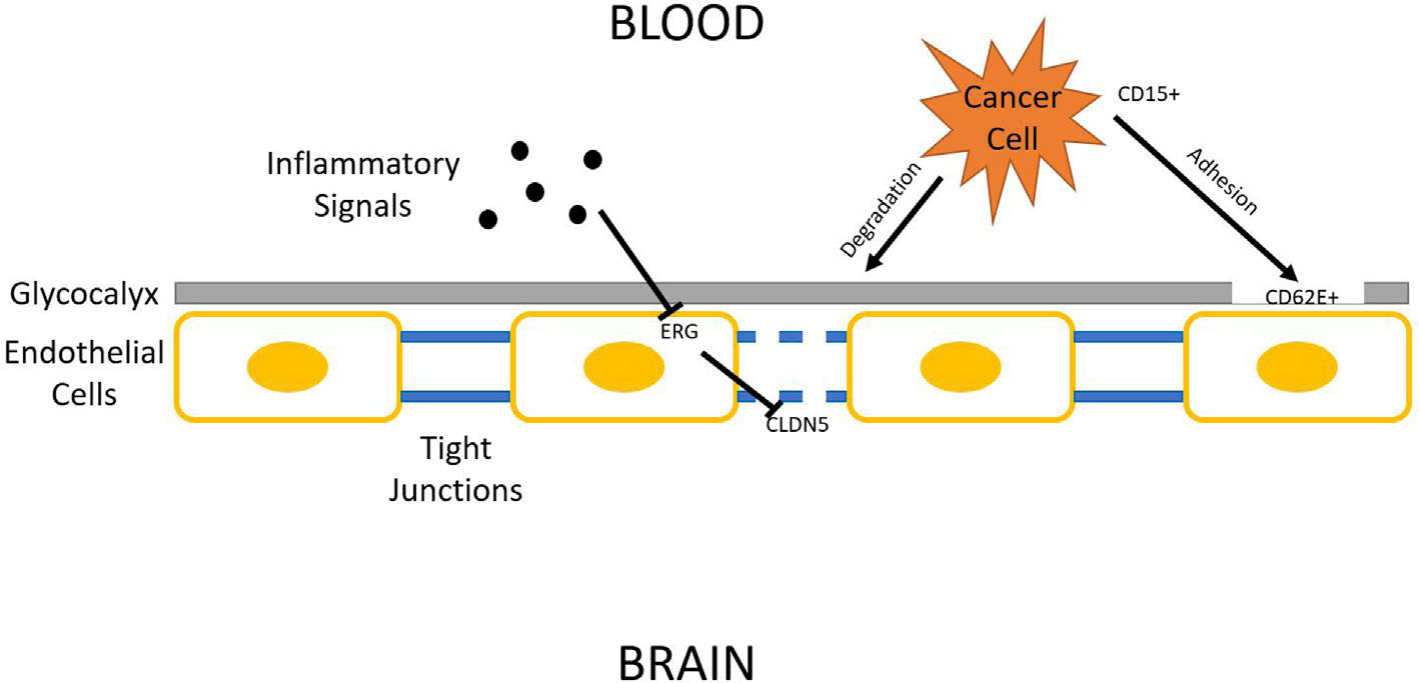
Blood–brain barrier (BBB) penetration. Lung cancer cells must breach BBB in order to access the brain parenchyma. The three general steps shown in this figure are glycocalyx degradation, epithelial adhesion, and tight junction downregulation.

Regardless of the strategy lung tumor cells use to adhere to brain endothelium, it now must breach it in order to get access to brain tissue. The endothelium, being part of the blood-brain-barrier (BBB), relies on tight junctions (TJs) between endothelial cells to provide an isolated environment for the brain. Occludin, claudins, and junctional adhesion molecules (JAMs) are all proteins present in TJs (67). Among those proteins, claudin–5 (CLDN5) is marked as pivotal in regulating BBB permeability. Reinforced expression of CLDN5 enhances BBB integrity and hinders invasion of lung adenocarcinoma A549 cells (68). Some mechanisms have been proposed to explain the downregulation of CLDN5 expression frequently found associated with lung cancer brain metastasis. One study identifies CLDN5 as a downstream target of ETS-related gene (ERG). ERG normally reduces endothelial permeability, but its function can be repressed by inflammatory signals, which could be secreted into circulation by tumors. ERG repression results in CLDN5 downregulation, increasing BBB permeability (69). One such inflammatory signal has been identified. Inflammatory cytokine TNF-α acts through NFκB signaling to downregulate CLDN5 expression (70). A multitude of natural product extracts, such as terpenes and phenolic compounds, were shown to be TNF-α inhibitors (71). Asiaticoside, a triterpenoid, enhances endothelial integrity of blood vessels, reducing their permeability through inhibiting TNF-α (72) (**Figure 2**).

After tumor cells breach the BBB, they enter the brain parenchyma densely packed with astrocytes and patrolled by microglia. Similar to their tactics used in invading lung tissues, tumor cells either evade or cooperate with local cell types. Astrocytes, glial cells specialized in nourishing neurons and maintaining ECM, secrete plasmin which suppresses brain metastasis. Tumors cells from metastatic lung cancers are found to express high levels of neuroserpins and serpin β2, which neutralizes death paracrine signals sent out by astrocytes (73). Astrocytes aid metastasis formation mainly through two methods: Substance secretion and gap junction interactions (74). A range of molecules secreted by reactive astrocytes (RAs) has been shown to correlate with lung cancer brain metastasis progression. Hyaluronic acid is found to promote lung cancer cell growth through the activation of protein kinase B (AKT)/mitogen-activated protein kinase (MAPK) (75). Endothelin-1 (ET-1) also activates the AKT/MAPK pathway. Its expression is enhanced in astrocytes by the upregulation of IL-6 and IL-8 expression in tumor cells (76). Polydatin(PD) inhibits MAPK, PI3K/AKT, and NF-κB pathways, and can also inhibit certain pro-inflammatory cytokines, such as TNF-α, IL-4, IL-1β, and IL-8 (77). Benzyl sulforaphane and Macrolactin F show potential in suppressing AKT/MAPK pathway in the liver and the bone, respectively (78, 79). Previous research also confirms that RAs produce IL-6, tumor necrosis factor-α (TNF-α), and IL-1β, all of which promote lung cancer brain metastasis (80). In another study, RAs secrete interferon-α (IFN-α) and tumor necrosis factor (TNF), which activates the STAT1 and NF-κB pathways in lung cancer cells residing in the brain (81). Astrocytes can also directly alter its ECM under influence of cancer cells. MMP-2 and MMP-9 secreted by astrocytes are found responsible for facilitating lung and breast cancer brain metastasis progression, presumably by making the EMC more favorable for tumor cells (82). Gap junctions formed between RAs and tumor cells also facilitates tumor growth. Survival genes such as GSTA5, BCL2L1, and TWIST1 are found to be upregulated in lung tumor cells that have formed gap junctions with astrocytes. The upregulation of these genes increases the chance of tumor cell survival and grants resistance to chemotherapy (83). The mechanism behind such regulation *via* gap junction is revealed in a 2016 study, which discovered that small RNA (sRNA) is tranferred between cells through gap junctions, marking sRNA as possible agents released by astrocytes that upregulates survival genes in tumor cells (84).

**Figure 3:**
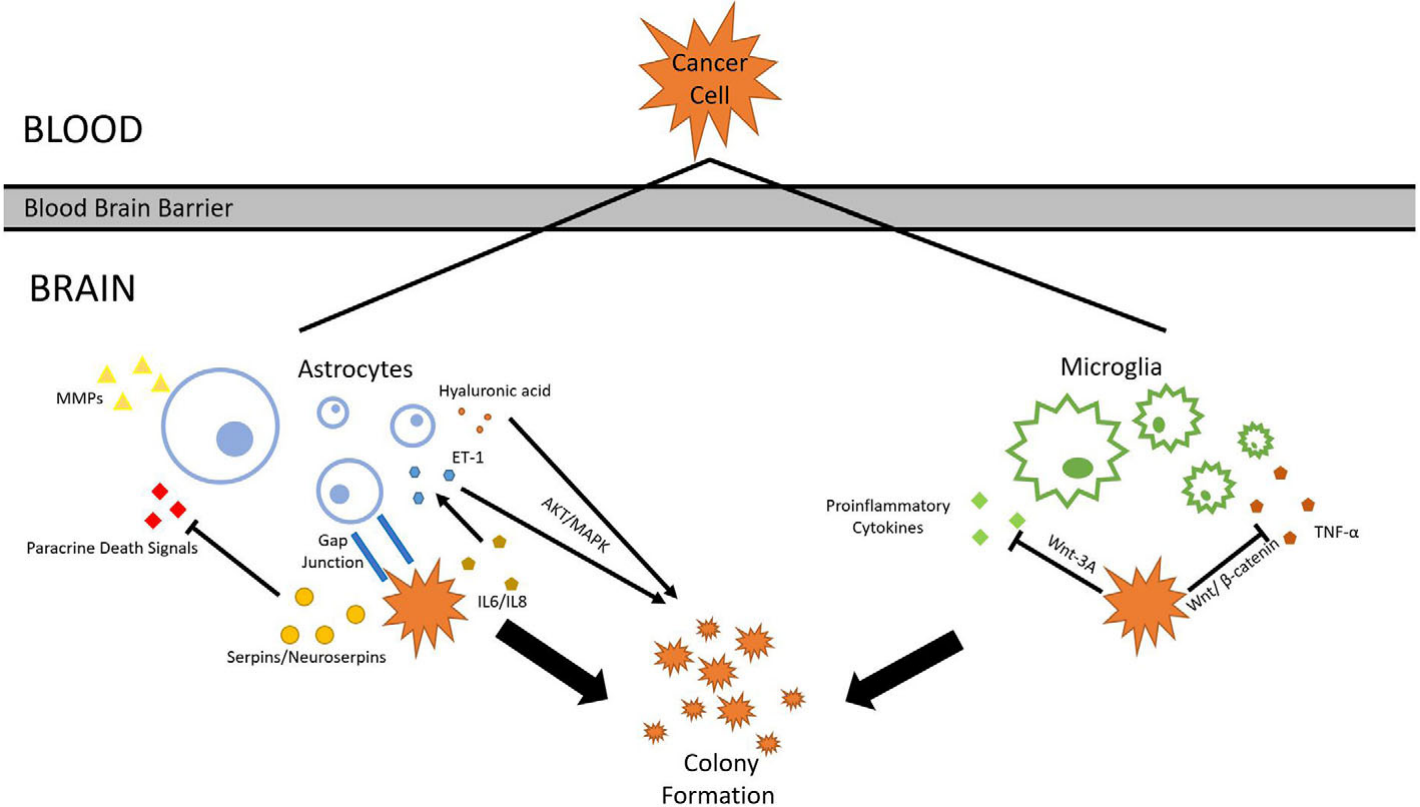
Astrocyte and microglia. To survive and colonize the brain, lung cancer cells must interact with surrounding glial cells such as astrocytes and microglia. Through various mechanisms, cancer cells are able to withstand the threats and cooperate with glial cells.

Similar to astrocytes, microglia, immune cells found exclusively in the central nervous system, are also capable of both tumorigenic and cytotoxic effects. Microglia can be broadly divided into pro-inflammatory and anti-inflammatory subtypes. Proinflammatory microglia suppress metastasis formation, while its counterpart increases metastatic tumor burden (85). Lipopolysaccharide (LPS)-activated microglia are cytotoxic to lung cancer cells by inducing apoptosis in them (86). Its activation is also correlated with increased production of proinflammatory cytokines (87). Cancer cells counteract the threat posed by microglia by suppressing its pro-inflamotory properties. Studies show that Wnt pathways are central to the regulation of microglial functions. Some Wnt pathways were once considered proinflammatory and thus unfavored for tumor growth. A 2011 study suggests that Wnt-3A stimulation triggers IL-6, IL-12, and tumor necrosis factor α production in microglia, which are all strong proinflammatory factors (88). However, multiple subsequent studies reach differing conclusions. Wnt-3A pathway is found to trigger the induction of M2 phenotype in microglia, which are anti-inflammatory and favor tumorigenesis (89). It is recognized as an oncogene, and its activation is correlated with Wnt-catenin pathway. Knocking down Wnt-1 and Wnt-3A suppresses tumor proliferation (90). Other than Wnt-3A, Wnt-5A has also been shown to correlate with increased presence of TAMs, though the effect of WNT-5A seems to be also pro-inflammatory (91). This complication of Wnt-3A and Wnt-5A effects can be potentially explained by a 2013 study. While Wnt-3A and WNT-5A alone upregulate proinflammatory responses, the combination of Wnts and LPS serves anti-inflammatory functions (92). Tumor cells likely utilize this characteristic to favor their own growth by upregulating multiple Wnt pathways, amplifying the anti-inflammatory and tumorigenic effects of microglia. Furthermore, Wnt/β-catenin signaling pathway, which has been discussed earlier in section 1, play a pivotal role in microglia function in brain metastasis. Through Gastrodin mediation, Wnt/β-catenin signaling pathway can downregulate multiple inflammatory factors such as TNF-α (93). Given that Wnt/β-catenin interaction is highly pronounced in lung cancer cells, lung cancer cells might gain an advantage by using this interaction to cooperate with microglia, resulting in higher metastatic potential in the brain. Secreted frizzled-related proteins (Sfrps) demonstrate differential inhibition of Wnt-3a activity (94). A study in 2011 found that SFRP-1 and the SFRP-like molecule V3Nter can inhibit β-catenin-activated tumor cells growth *in vivo*, though another study argues that sFRPs have dual effects on Wnt/β-catenin signaling depending on various factors (95, 96). With more research currently underway, much more will be uncovered about microglia’s role in lung cancer brain metastasis in the future (**Figure 3**).

#### Bone

At 39%, bone metastasis is the most frequent site of metastasis of adenocarcinoma. The presence of bone metastasis also signals worse prognosis compared to other metastasis sites (59). Multiple homing mechanisms have been revealed in lung cancer that metastasizes to the bone. In breast and prostate cancers, stromal cell-derived factor 1 (SDF-1) and its receptor CXCR4 are responsible for attracting tumor cells to the bone marrow (97). This coincides with a study recently published in 2019, which observed increased expression of CXCR4 in lung carcinoma (98). It is not impossible that the overexpression of CXCR4 in lung cancer cells enabled better attachment of tumor cells to the bone. Kisspeptin-10 (KP-10) can inhibit tumor cell invasion and EMT induced by SDF-1 through down-regulating CXCR-4 expression (99). Recent studies also reveal that certain CXCR4-targeting nanobodies inhibit CXCR4-related functions, which may yet unravel another treatment option (100). With striking resemblance to the aforementioned CXCR4/SDF-1 homing mechanism, CXCL10/CXCR3 also emerge as a potential homing mechanism used by lung adenocarcinoma. A 2012 study demonstrates that CXCL10 attracts CXCR3-expressing tumor cells to the bone, though omitting its connection with lung cancer (101). A French team discovered that a CXCL10/CXCR3-A autocrine loop exists in invasive mucinous adenocarcinoma (IMA), in which CXCL10 upregulates CXCR3-A (102). It is thus possible that CXCL10 in the bone stroma may upregulate CXCR3 production in lung cancer cells, which in turn became more attracted to the bone. One confirmed homing mechanism used by lung adenocarcinoma cells is discoidin domain receptor 1 (DDR1). DDR1 is a cell surface receptor that can bind to several types of collagens and enhance cell adhesion. It was observed back in 2010 that DDR1 upregulation is a frequent occurrence in invasive lung adenocarcinoma. Its upregulation is correlated with increased tumor invasiveness and worse prognosis, although the precise mechanisms were elusive (103). The upregulation of DDR1 is found to be inducible by collagen I, which is a major component of osteons in the bone (104). Lung cancer cells may express DDR1 when induced by collagen I in the bone, which completes its homing process. Disrupting DDR1 pathway significantly reduced tumor-bone engagement, lowering cancer cell’s potential to metastasize to the bones (105), and multiple means of doing so has been discovered. Tetrahydroisoquinoline-7-carboxamide based DDR1 inhibitor 7ae developed to treat acute lung injury (ALI) bind tightly to DDR1, reducing its kinase activity (106). Other studies reveal that celastrol downregulates E2F1 (107), while E2F1 silencing downregulates the expression of DDR1 (108). However, these studies are primarily concerned with osteosarcomas and hepatocellular carcinoma, therefore their existence in lung cancer bone metastasis awaits further validation.

Maintenance of bone tissues requires cooperation between osteoblasts (OBs) and osteoclasts (OCs). Cancer cells often hijack either OB or OC, forming osteoblastic or osteolytic lesions in bone tissues. Since osteoblastic lesions are infrequent in NSCLC bone metastasis, it will not be discussed in this article. In osteoclastic bone metastasis, which is most common in NSCLC, a “tumor-OC cooperation” is found. Tumor cells secrete pro-osteoclastogenic substances, while osteolysis generates substances that benefit tumor growth (109). One initiator of such a vicious circle is RANK ligand (RANKL). RANKL has been demonstrated to be essential to osteolytic activities induced by NSCLC cells. The inhibition of RANKL reduced osteoclastogenesis stimulated by NSCLC cells, and metastasis progression is slowed, possibly due to fewer growth factors yielded from osteolysis (110). A 2018 study confirms this discovery using mouse models. RANKL expression is found in NSCLC in primary lung cancer, and it is correlated with enhanced tumor growth (111). It is thus possible that NSCLC tumor cells present in the bones express RANKL, which enables osteolytic activities. With such a critical role in bone metastasis formation, RANKL is by far one of the most accurate predictors of bone response in patients with bone metastasis (112). Multiple factors that are associated with RANKL expression in lung tumor cells have also been identified. Thrombospondin (TSP)-2 knockdown is correlated with the inhibition of osteolytic metastasis of lung cancer. It is proposed that TSP-2 enhances osteoclastogenesis through RANKL dependent pathways (113). RANKL can also upregulate Basigin-2, which induces MMPs and VEGF expression, contributing to lung cancer bone metastasis through osteoclastogenesis (114). Extensive research has yielded numerous treatments that target RANKL, most of which are conducted quite recently. Estrogens and androgens are shown to inhibit OB-driven osteoclastogenesis (115). A myriad of drugs, such as Baricitinib, Matrine, and Sciadopitysin all suppress RANKL-mediated osteoclastogenesis (116–118).

With extensive osteolysis underway, a range of tumor trophic substances will be released, one of which being transforming growth factor beta (TGFβ). TGFβ is dysregulated in malignant cells, including lung cancer, and upregulation of TGFβ in the ECM of lung cancer promotes NSCLC progression and invasion (119). Not only does TGFβ promote tumor cell proliferation, it also contributes to the vicious cycle by encouraging osteolysis. TGFβ upregulates Gli2, which in turn promotes osteolysis through increased secretion of osteolytic factors such as parathyroid hormone-related protein (PTHrP) (120). In osteoclastic bone metastasis, micro-RNAs (miRNAs) also play an important role, acting as key regulators in bone metastasis progression. Most miRNAs discovered are found to be inhibitory, and very few are shown to be pro-metastasis. One such miRNA is miR-326, which is found to correlate strongly with tumor burden in lung cancer bone metastasis (121). Serum microRNA-139-5p expression in mesenchymal stem cells (MSCs), progenitors of OBs, is sharply reduced when exposed to lung cancer cells A549 and L9981. The downregulation of microRNA-139-5p is correlated to increased osteolysis (122). miR-33a, which suppresses bone metastasis, is downregulated in lung cancer cells. Decreased miR-33a expression is correlated with increased PTHrP expression and RANKL production, both of which contribute to osteolysis (123). In conclusion, NSCLC cells manipulate OCs through secretion of pro-osteoclastogenesis substances and regulation of various miRNAs, allowing it to form bone metastasis.

Besides osteoclasts, adipocytes and macrophages also participate in bone metastasis formation. Adipocytes store fat, which can be used by lung cancer cells as an energy source. Compared to non-malignant lung cells, lung cancer cell A549 has twenty times more lipid droplets in its cytoplasm, suggesting that it is capable of using lipid as an alternative energy source to glucose (124). When fatty acid production *in vivo* or *in vitro* is inhibited, NSCLC growth is also hindered, suggesting cancer cell’s reliance on fatty acid synthesized and stored in adipocytes (125). Cancer-associated adipocytes (CAAs) release fatty acids through lipolysis, while cancer cells can harvest the energy in them using β-oxidation (126). Moreover, CAAs can produce leptin, which has been demonstrated to promote EMT changes in A549 lung cancer cells, contributing to tumor cell mobility and consequently their invasiveness in the bone (127). Macrophages are also correlated with bone metastasis. A reduction in the number of macrophages is accompanied by the inhibition of bone metastasis (128). Rac2, a small GTPase, controls M1 to M2 differentiation in macrophages, which suppresses inflammatory responses (129). IL-13-induced phosphorylation of STAT6 has also been identified as a major pathway leading to macrophage M2 polarization (130). Furthermore, bone marrow macrophages (BMMs) are the major source of cathepsin K (CTSK), which promotes tumor progression in bones (131). Although substantial progress is made regarding the roles of adipocytes and macrophages in bone metastasis, very few researches have explored their interactions with lung cancer cells in particular. That being said, more effort should be invested in clarifying the connections between adipocytes, macrophages, and invading lung cancer cells. In essence, lung cancer bone metastasis involves a unique homing mechanism, a osteoclastic invasion, and diverse tumor-bone interactions.

#### Liver

Compared to NSCLC metastasis in the brain and the bone, metastasis in the liver is less frequent, but still at a high occurrence. It is estimated that 16.7% of metastasized NSCLC patients develop liver metastasis (132). With an average median survival time of 4 months, liver metastasis in NSCLC confers a worse prognosis than lung, brain, and bone metastasis according to a recent study (133). NSCLC liver metastasis often predicts poor progression-free survival with treatments underway, including Nivolumab (134) and erlotinib targeted therapy (135).

When tumor cells enter the liver through the hepatic artery or portal vein, they will encounter the sinusoid, which endothelium is composed of liver sinusoidal endothelial cells (LSECs). LSECs have dual roles in metastasis formation. When tumor cells obstruct the sinusoid, inflammatory response will be triggered by LSECs, leading to production of various substances inhibiting metastasis (136). Among these substances is TNF-α, capable of inducing apoptosis in tumor cells. Lung cancer cells, however, often express phenotypes that resist the effect of TNF-α. A study dating back to 2000 suggests that NF-κB activation increases lung cancer cells’ resistance to TNF-α (137). Cylindromatosis (CYLD) is considered to positively correlate with the apoptotic effect of TNF-α. Its expression in lung cancer cells is low, suggesting that lung cancer cells may downregulate CYLD in order to prevent TNF-α induced apoptosis (138). The effect of TNF-α can only be realized when it binds to TNF-α receptor (TNFR1), which contains TNFR1 promoter -223/-29 in lung cancer cells. The downregulation of TNFR1 is associated with decreased effectiveness of TNF-α, pointing to a potential mechanism lung cancer cells may use to evade TNF-α (139). Tumor cells may even benefit from exposure to TNF-α. One study concluded that through the upregulation of MMP-13, TNF-α promotes tumor growth in lung cancer cells (140). Significant progress is made to inhibit cancer growth through manipulating TNF-α expression. Highly N-acetylated COS (NACOS) inhibits TNF-alpha-induced E-selectin expression in ECs *via* the JNK/NF-κB pathways, potentially attenuates tumor cell adhesion with ECs (141). A research conducted in 2013 proposed that Acacetin can also inhibit TNF-α-induced E-selectin expression, as well as the activation of NF-κB by TNF-α (142). ZLJ-6 inhibits a range of adhesion molecules including E-selectin, ICAM-1, and VCAM-1, and, although it was once believed that the COX/5-LOX pathway underlies its effect, was found to be independent of this pathway and is rather mediated by NF-κB (143).

Other than TNF- α, LSECs also produce IFN-γ. IFN-γ induces extracellular trap cell death (ETosis) in A549 lung cancer cells. Reactive oxygen species (ROS), another substance LSECs secrete, regulates IFN-γ-induced mimic ETosis in lung cancer cells (144). This mechanism, however, has been found to be defective in many lung cancer cells. PC14PE6/AS2 human lung cancer cells were shown to be unsusceptible to IFN-γ-induced autophagy (145). PC14PE6/AS2 human lung adenocarcinoma cells are presented with reduced responsiveness in IFN-γ signaling, possibly due to natural PTEN loss. PTEN silenced A549 cells also demonstrate reduced susceptibility to IFN-γ through ROS/SHP2 signaling (146). It is also suggested that IFN-γ alone may not be able to inhibit lung cancer proliferation, as only PD-L1+ lung carcinomas are affected by IFN-γ. Thus PDL1- lung cancer cells may be immune to IFN-γ induced ETosis (147). Possible mechanisms have been proposed. IL-10 and IL-10R are found to be overexpressed in cells surrounding NSCLC cells in the lungs, and confer resistance to IFN-γ through regulation of the PD1/PD-L1 pathway, although such phenomenon has not been studied in the liver (148). LSECs contribute to the formation of liver metastasis by expressing various adhesion molecules. Early studies have shown that the presence of tumor cells in hepatic circulation can trigger a rapid induction of E-selectin expression in LSECs, which could serve as adhesive molecules that facilitate tumor cell colonization (149). Blocking E-selectin expression causes a 97% reduction in liver metastasis compared to the control group (150). Tumor growth is also suppressed when E-selectin expression in LSECs is inhibited through the prevention of angiogenesis, suggesting E-selectin’s importance in metastasis progression in the liver (151). Vascular cell adhesion molecule-1 (VCAM-1) is also found expressed in LSECs, which participates in tumor-stromal interactions (152). Downregulation of VCAM-1 is correlated with suppressed lung cancer cell growth (153), while VCAM-1 knockdown reduces A549 cells’ ability to migrate (154).

Furthermore, since LSECs are fenestrated, tumor cells can directly interact with the basement membrane underneath (155). One type of the receptors present on the surface of basement membrane matrix is integrin receptors (156). Integrins αv, α5, β1, β3, and β5 are shown to promote survival and metastasis of lung cancer cells through either providing mechanical attachment to ECM, or the generation of various cell signals stimulating metastasis formation (157). Suppressing metastasis-related integrins such as integrin αv and integrin β3 in lung cancer inhibits motility of tumor cells (158). Interestingly, nitric oxide (NO), which is released by LSECs, is demonstrated to increase the expression of integrin αv and β1 in three types of NSCLC cell lines. Focal adhesion kinase, active RhoA, and active cell division control 42, all of which are migration associated proteins, are downstream targets of these two integrins (159). Integrin β4 is also found overexpressed in NSCLC cells, which increases venous invasion and correlates with a poor prognosis (160). Inhibiting integrin expression may serve as a new therapeutic strategy to suppress liver metastasis. E3 ubiquitin ligase Smurf1, for example, was demonstrated to be an effective inhibitor of integrin expression (161).

After tumor cells extravasate from sinusoid, they will encounter hepatocytes, the main cells of the liver. There has been evidence pointing to the presence of tumor cell-hepatocytes interactions. Claudin-2 level is elevated in breast cancer liver metastasis, and it is responsible for adhering breast cancer cells to hepatocytes (162). Although such interaction is not yet studied between lung adenocarcinoma cells and hepatocytes, there are clues that suggest the existence of this interaction. Claudin-2 is highly expressed in lung adenocarcinoma and is linked to increased tumor cell proliferation (163), and another study in the same year also concluded that decreased claudin-2 hinders lung carcinoma development (164). Therefore, it is not impossible that lung cancer cells may employ a similar technique to better adhere to hepatocytes. Hepatocytes also alter E-cadherin expression in cancer cells. E-cadherin expression is critical in EMT and EMrT, which are essential in metastatic processes. p0071/E-cadherin interaction in lung cancer cells is shown to increase their metastatic potential (165). The downregulation of E-cadherin in A549 cells induces cancer stem cell properties, which are necessary for metastasis. The upregulation of E-cadherin, in contrast, causes cancer stem cells to diminish (166). Therefore, when lung cancer stem cells reach liver tissues, they must upregulate E-cadherin levels to perform EMrT, which trades their stem cell features with epithelial features, invasiveness. Hepatocytes have the ability to upregulate E-cadherin expression in tumor cells in prostate cancer liver metastasis, which seemed to be regulated by lowering epidermal growth factor receptor (EGFR) signaling (167). Since EGFR mutation is relatively common in lung cancer, this mechanism found between prostate cancer cells and hepatocyte may also be used by lung cancer cells, although currently it is not thoroughly studied.

### New Treatment Strategy and Molecular Mechanisms of NSCLC Metastasis

In recent years, many new drugs have been put into clinical trials for the treatment of metastatic NSCLC to verify their toxicity and efficacy to enrich the treatment first-line options for patients. Several carcinogenic drivers including EGFR have been identified and studied. The development of new therapies, including targeted therapy and immunotherapy, has shown encouraging results in prolonging the survival of patients with NSCLC. For example, inhibitors targeting the PD-1/PD-L1 immune checkpoint have been developed as an effective immunotherapy for metastatic NSCLC. In NSCLC cells, the binding of PD-1 and PD-L1 promotes T-cell tolerance and escape from host immunity. Pembrolizumab, nivolumab, and cemiplimab are anti-PD-1 inhibitors, and atezolizumab, durvalumab, and avelumab are anti-PD-L1 inhibitors (168). PD-1 inhibitors offer enhanced survival benefits and fewer adverse events than PD-L1 inhibitors (169). As mentioned above, any malignant transformation of EGFR may lead to the spread of lung cancer (170), so EGFR as a target has become the main research scheme to inhibit the metastasis of NSCLC. For example, from 2003 to 2019, first-generation drugs (erlotinib, gefitinib), second-generation drugs (afatinib, dacomitinib), third-generation drugs (osimertinib, rociletinib), combination therapy (docetaxel+pemetrexed), and fourth-generation drugs (EAI001 and EAI045) were successively developed (171). Although many drugs have been proved to be effective in clinical trials, some patients will have drug resistance and complex toxicity after a period of treatment. Therefore, it is still necessary to further study the molecular mechanism of NSCLC metastasis and explore potential biomarkers for the development of new therapeutic strategies

HER2 overexpression has been observed in 3%–38% of NSCLC, while strong HER2 protein overexpression is found in 2.5% of NSCLC (172). Leeza Shrestha et al*.*, designed a peptidomimetic(compound 18) that binds to domain IV of the HER2 receptor, disrupts the homo/heterodimerization of HER2, and inhibits downstream signaling for cellular proliferation and growth, hence, can be used as a potential therapeutic agent for the treatment of NSCLC (173). A study from China suggests that traditional Chinese herbal medicine may play a role in antimetastasis of malignant tumors. Chinese herbal medicine Wenxia Changfu formula reverses cell adhesion-mediated drug resistance in lung cancer. Xiaoai Jiedu recipe can inactivate p38 MAPK signaling pathway in NSCLC cells to inhibit its proliferation and metastasis (174). Recent studies have found that CERS6 has important roles in lung cancer migration and metastasis, which proves that the miR-101-CERS6 pathway can be targeted, with potential benefits provided for affected patients (175). High expression of monoamine oxidase A (MAOA) in NSCLC is related to EMT and the development of clinicopathological features of NSCLC (176). The potential MAOA inhibitor G11 may inhibit paclitaxel‐resistant NSCLC metastasis and growth by impacting on p‐AKT, VEGF, HIF1α, and MMP2 or MMP9. These findings have established MAOA as a promising therapeutic target in drug-resistant NSCLC and provide a feasible method of MAOA-targeted combined therapeutics (177). The combination of chemical and mechanical signals should also be considered in the new direction of drug development. Baicalein inhibited the formation of protrusive structures and leader cells by the combined effects that decreased both ezrin S-nitrosylation (chemical signaling) and ezrin tension transduction (mechanical signaling), and hampered NSCLC invasion and metastasis. Future sudy and improved clinical prospects for patients with NSCLC will depend on continued focus on combination of basic discovery and clinical translational research.

## Discussion and Perspective

As showcased in this review, a substantial amount of effort has already been devoted to unraveling mechanisms behind NSCLC’s high metastasis potency as well as the wide range of potential organs for colonization that make NSCLC stand out from the majority of cancers. The chronological distribution of the references indicates that discoveries in this field have been made at an ever-increasing pace. Through studying the numerous molecular pathways involved in NSCLC metastasis, we are starting to find patterns that may one day fit into the puzzle of NSCLC metastasis containment and management. Many novel pathways identified to be essential to NSCLC metastasis had yielded new treatment options, and many of these targeted therapies were shown to be effective in restricting NSCLC metastasis. Still, for every question clarified, dozens of confusions arise. As demonstrated by this extensive review, lung cancer metastasis is an extremely complicated event and is further complicated by the type of lung cancer, metastatic organ, and more. Here, we list out some inquiries and topics that may be worth further investigation, including the limitations and unsolved enigmas of current research. Advances made in these regards could be critical to our current knowledge base of NSCLC metastasis.

- In many organs studied for cancer metastasis, such as the bone, very few researches studied lung cancer metastasis in these organs. As a result, extrapolation might be frequently used in an attempt to apply progress in other cancer types to NSCLC. These inferences are usually made by finding similarities between the discussed cancer types and lung cancer, and then suggest that lung cancer could utilize a similar mechanism. These inferences, being extrapolations, have the factor of conjecture. In order to clarify whether these suggestions are valid in NLCSC, we encourage more studies to be done to either validate or dismiss the existence of these mechanisms.
- Cancer progression can be viewed as a failure of the immune system to identify and eradicate cancer cells. Thus, many immune cells are associated with cancer, some actively involved in cancer invasion and metastasis. Each part of the human body has a unique blend of different immune cells, such as BMM in the bone and microglia in the brain. Most of the works currently available explain how cancer cells evade a certain immune cell’s immune response. Few works, however, studied whether one mechanism cancer cell used to fend off a certain type of immune cell might be involved in another interaction between cancer cells and a different type of immune cell. Since there are plenty of overlaps between the molecules secreted by or the signal pathways involved in different immune cells, it may be worth the effort to identify these overlaps to discover treatment options that can simultaneously attack multiple tumor cell-immune cell interactions, yielding a better prognosis.
- When tumor cells arrive at a distant organ, it can instantaneously spawn a new metastasis, but it can also enter a period of dormancy and become activated at a later time. The latter have been first discovered in the bone marrow, but it is now evident that many organs including the liver can house dormant tumor cells. These dormant cells can survive chemotherapy since it is not hyper-proliferating nor over metabolically active, thus they may spawn new metastasis even though all visible tumors have been eradicated. This mechanism has been extensively studied in the bone, but fewer studies are devoted to other organs such as the liver, and even few of them use NSCLC as their cancer type. A better understanding of this mechanism can be promising to the invention of therapies that prevent cancer recurrence.

Looking at only the tip of an iceberg of NSCLC metastasis, there is obviously still a quite formidable amount of knowledge to be discovered. However, with the progress made with high pace fueled by new generations of biotechnology, preventing NSCLC metastasis, or even curing NSCLC, may became reality in the future not far away.

## Author Contributions

TZhu, XB, MC, RL, JZ, TZhe, and KX wrote the original draft. TZhu, XB, WZ, and SZ wrote, reviewed, and edited the manuscript. All authors contributed to the article and approved the submitted version.

## Funding

This work was supported by the Shanghai Municipal Science and Technology Commission (grant number: 19441904500) and the China Postdoctoral Science Foundation (2019M651376). This work was supported in part by the National High Technology Research and Development Program of China (2015AA020104), the National Natural Science Foundation of China (31471239 and 31671368), and the 111 Project (B13016).

## Conflict of Interest

The authors declare that the research was conducted in the absence of any commercial or financial relationships that could be construed as a potential conflict of interest.

## Abbreviations

AKT, activation of protein kinase; ALI, acute lung injury; APC, antigen-presenting cell; BBB, blood–brain barrier; BMM, bone marrow macrophages; CAA, cancer-associated adipocytes; CAM, cellular adhesion molecules; CLDN5, claudin-5; CTSK, cathepsin k; CYLD, cylindromatosis; DC, dendritic cells; DDR1, discoidin domain receptor 1; ECM, extracellular matrix; EGFR, epidermal growth factor receptor; ERG, ETS-related gene; EMT, epithelial–mesenchymal transition; ETosis, extracellular trap cell death; FASN, fatty acid synthase; HIF, hypoxia inducible factor; HLA-E, human leukocyte antigen e; IFN, interferons; IL, interleukins; IMA, invasive mucinous adenocarcinoma; JAM, junctional adhesion molecules; KP-10, kisspeptin-10; LPS, lipopolysaccharide; LSEC, liver sinusoidal endothelial cells; MF, myofibroblasts; miRNA, Micro-RNAs; MAPK, mitogen-activated protein kinase; MMP, metalloproteinases; MSC, mesenchymal stem cells; MAOA, monoamine oxidase A; NACOS, N-acetylated COS; NSCLC, non-small cell lung cancer; NK, natural killer cells; NO, nitric oxide; OBs, osteoblasts; OC, osteoclasts; PD, polydatin; PDL1, programmed death-ligand 1; PD1, programmed death receptor 1; p-PTEN, phosphorylation of the PTEN C-terminus; PGE2, prostaglandin E2; PTHrP, parathyroid hormone-related protein; RA, reactive astrocytes; ROS, reactive oxygen species; RANKL, RANK ligand; SCFA, short-chain fatty acids; SDF-1, stromal cell-derived factor 1; SNX9, nexin 9; Sfrps, secreted frizzled-related proteins; sRNA, small RNA; TADC, tumor-associated DCs; TAM, tumor-associated macrophages; TGF, tumor growth factors; TJ, tight junctions; TME, tumor microenvironment; TNF, tumor necrosis factors; Tregs, regulatory T cells; TSP, thrombospondin; VCAM-1, vascular cell adhesion molecule-1; VEGF, vascular endothelial growth factors; YY1, yin yang 1.
